# Two high-rate pentose-phosphate pathways in cancer cells

**DOI:** 10.1038/s41598-020-79185-2

**Published:** 2020-12-17

**Authors:** Vanessa Cossu, Marcella Bonanomi, Matteo Bauckneht, Silvia Ravera, Nicole Righi, Alberto Miceli, Silvia Morbelli, Anna Maria Orengo, Patrizia Piccioli, Silvia Bruno, Daniela Gaglio, Gianmario Sambuceti, Cecilia Marini

**Affiliations:** 1 Department of Health Sciences, University of Genoa, Via Antonio Pastore 1, 16132 Genoa, Italy; 2 Department of Biotechnology and Biosciences, University of Milano-Bicocca, Piazza della Scienza 2, 20126 Milan, Italy; 3 ISBE. IT/Centre of Systems Biology, Piazza della Scienza 4, 20126 Milano, Italy; 4 IRCCS Ospedale Policlinico San Martino, UO Nuclear Medicine, Largo Rosanna Benzi 10, 16132 Genoa, Italy; 5 Department of Experimental Medicine, University of Genoa, Via de Toni 14, 16132 Genoa, Italy; 6 Cell Biology Unit, IRCCS Ospedale Policlinico San Martino, Largo Rosanna Benzi 10, 16132 Genoa, Italy; 7 Institute of Molecular Bioimaging and Physiology (IBFM), National Research Council (CNR), Via Fratelli Cervi 93, 20090 Segrate, Italy

**Keywords:** Cancer metabolism, Metabolomics

## Abstract

The relevant role of pentose phosphate pathway (PPP) in cancer metabolic reprogramming has been usually outlined by studying glucose-6-phosphate dehydrogenase (G6PD). However, recent evidence suggests an unexpected role for a less characterized PPP, triggered by hexose-6-phosphate dehydrogenase (H6PD) within the endoplasmic reticulum (ER). Studying H6PD biological role in breast and lung cancer, here we show that gene silencing of this reticular enzyme decreases cell content of PPP intermediates and d-ribose, to a similar extent as G6PD silencing. Decrease in overall NADPH content and increase in cell oxidative status are also comparable. Finally, either gene silencing impairs at a similar degree cell proliferating activity. This unexpected response occurs despite the absence of any cross-interference between the expression of both G6PD and H6PD. Thus, overall cancer PPP reflects the contribution of two different pathways located in the cytosol and ER, respectively. Disregarding the reticular pathway might hamper our comprehension of PPP role in cancer cell biology.

## Introduction

Cancer metabolism is characterized by high consumption of glucose and its incomplete breakdown to lactate despite a normal oxygen tension. Trying to explain the mechanisms underlying this “Warburg effect”, many recent studies reported a relevant contribution for the pentose phosphate pathway (PPP) due to its function in the recognition of both intracellular and extracellular signals^1,2^. This consideration fits the versatile role of this pathway and the dual-step nature of its reactions sequence. Indeed, the first PPP oxidative phase is selectively accelerated under redox stress to increase the NADPH equivalents for antioxidant responses. By contrast, PPP acceleration extends to the non-oxidative phase when the high proliferating activity asks to combine bio-reductive syntheses, and thus high NADPH levels, with large amounts of d-ribose-5-phosphate (R5P) for the production of RNA, DNA, nucleosides, ATP, coenzyme A and other coenzymes such as NADH, FADH_2_ and NADPH^3^.

Since its discovery by Warburg in 1931^4^, PPP is considered a cytosolic pathway whose rate-limiting step is catalyzed by glucose-6P-dehydrogenase (G6PD). Thus, most studies focused on this enzyme to verify the potential PPP role in cancer metabolism and reported a mandatory function of G6PD activity for survival and proliferation of cultured cancer cells in vitro^1^. Nevertheless, the G6PD role in onset and progression of tumor lesions in living patients is markedly less obvious, since even severe G6PD deficiency (down to < 1% of normal activity) does not decrease cancer incidence^5,6^ and can even increase mortality for several cancer types^7^. According to these epidemiological observations, an alternative pathway should overcome the slow rate of cytosolic PPP in providing the high amounts of NADPH and R5P needed by actively proliferating cells.

The endoplasmic reticulum (ER) of almost all eukaryotic cells contains an autosomic enzyme, the hexose-6-phosphate dehydrogenase (H6PD), that, differently from its cytosolic—sex-linked—counterpart G6PD, can oxidize a large number of phosphorylated and free hexoses (hence the alternative denomination of glucose dehydrogenase)^8–10^. Although the relevance of this reticular pathway in overall glucose degradation has been scarcely considered, recent studies documented that H6PD activity is enhanced in several cancer types (https://www.proteinatlas.org/ENSG00000049239-H6PD/pathology) and contributes to their proliferating activity and migratory potential^11^. Similarly, we recently reported that down-regulation of H6PD with short interfering short interfering RNA ((si)RNA) decreases proliferating activity in cell lines derived from murine breast and colon cancer^12^.

Although NADP reduction to NADPH can be warranted by several G6PD-independent pathways (mostly isocitrate dehydrogenase and malic enzyme), most intracellular R5P is produced by the conversion of Ribulose-5P (Ru5P) through the activity of the R5P isomerase in the first reaction of the non-oxidative PPP phase^13^. In the present study, we used a metabolomic approach to verify the role of H6PD in overall PPP activity in cancer cells, focusing our attention on d-ribose as final product of PPP characterized by only one recognized source^13^.

## Results

### Absent interference between G6PD and H6PD gene expression

**Figure 1:**
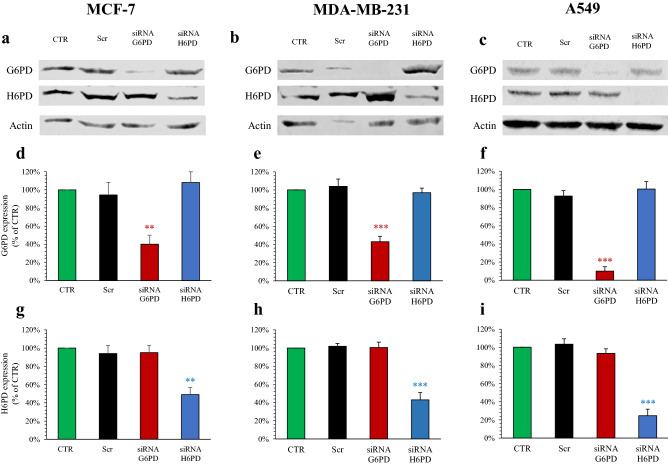
G6PD and H6PD Gene Expression after siRNA transfection. Representative images of western blot analysis of MCF-7 (**a**), MDA-MB-231 (**b**) and A549 (**c**) (run under the same experimental conditions for each cell line), reporting G6PD, H6PD and Actin expression. Relative densitometry analyses of G6PD and H6PD expression in MCF-7 (**d**,**g**), MDA-MB-231 (**e**,**h**) and A549 (**f**,**i**), under control condition (CTR, green), scramble siRNA (Scr, black), siRNA G6PD (red) and siRNA H6PD (blue). In all cell lines, scramble siRNA-transfected cells did not show significant variations in the levels of G6PD and H6PD with respect to control ones. G6PD siRNA delivery significantly reduced G6PD expression in MCF-7 (**d**), MDA-MB-231 (**e**) and A549 (**f**), while keeping unaltered H6PD expression. Similarly, H6PD siRNA transfection selectively reduced enzyme expression in MCF-7 (**g**), MDA-MB-231 (**h**) and A549 (**i**). Western blot analysis confirmed the efficiency and selectivity of gene silencing in all cancer cell models, documenting the absence of any interference between the trigger enzymes G6PD and H6PD. Data are expressed as % ± SD of respective control condition (n = 3). **p < 0.01, ***p < 0.001 vs corresponding scramble. The full-length gels are presented in Supplementary Fig. 1.

Experiments were performed in three human cell lines already tested for the relevance of PPP in proliferating activity^14,15^ and representative of human breast cancer either hormone-sensitive (MCF-7) or triple-negative (MDA-MB-231) as well as lung adenocarcinoma (A549)^16^, respectively. Silencing of either G6PD or its reticular counterpart H6PD was performed using specific siRNAs, whose effectiveness was confirmed by both real-time PCR (data not shown) and protein levels by western blot analysis (Fig. 1a–c). No significant cross-silencing was observed, in any of cell lines, confirming bioinformatics data of siRNA gene selectivity and, most importantly, documenting the absence of any biological interference between the relative expressions of the two PPP trigger enzymes, the one located within the cytosol and the other in the ER (Fig. 1d–i).

### Effect of G6PD and H6PD silencing on culture growth

**Figure 2:**
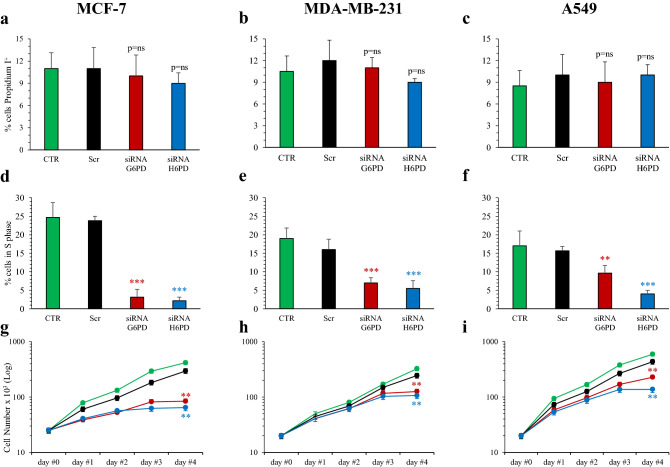
Effect of G6PD and H6PD silencing on culture growth. Percent of Propidium iodide positive dead cells in MCF-7 (**a**), MDA-MB-231 (**b**) and A549 (**c**) cultured under control condition (CTR, green), scramble siRNA (Scr, black), siRNA G6PD (red) and siRNA H6PD (blue). Either G6PD or H6PD silencing did not affect the number of dead cells. Percent of cells in S phase in MCF-7 (**d**), MDA-MB-231 (**e**) and A549 (**f**). Silencing of either G6PD or H6PD caused a marked reduction in the number of actively DNA synthetizing cells. Panels (**g**–**i**) display the time course of culture growth: silencing of either enzyme cause a progressive deceleration of cell proliferation that eventually reached a flat phase after 72 h. **p < 0.01, ***p < 0.001 vs corresponding scramble.

We thus evaluated the response of culture growth after gene silencing. Inhibition of either G6PD or H6PD expression virtually did not affect the percentage of dead cells, positive at propidium iodide staining (Fig. 2a–c). By contrast, % cells in S cell cycle phase, i.e. cells in the DNA replicative status, was markedly reduced in all cell lines by silencing of either G6PD or H6PD (Fig. 2d–f). This observation was coherent with the culture growth rate estimated by cell number increase: silencing of either G6PD or H6PD resulted in a progressive reduction of cell counts, that eventually reached an almost flat phase, in all three tumor lines (Fig. 2g–i). Given that cell mortality could not be responsible for the observed drop of S phase cell fraction, the data suggest that G6PD and H6PD silencing both affect the G1-S transition.

### Effect of G6PD and H6PD on overall cell PPP activity

We then adopted a metabolomic approach to compare the relative abundance of both lactate (as the terminal product of glycolysis) and the following PPP intermediates: 6-phosphogluconic acid (6-PGluc), Ru5P, R5P, xylulose 5-phosphate (X5P), sedoheptulose-7-phosphate (S7P) and D-glyceraldehyde 3-phosphate (GAD3P).

**Figure 3:**
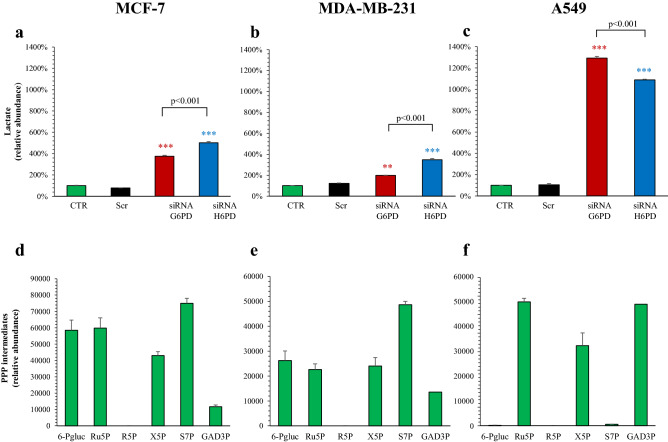
Lactate and PPP intermediates content. Relative lactate abundance in MCF-7 (**a**), MDA-MB-231 (**b**) and A549 (**c**) under control condition (CTR, green), scramble siRNA (Scr, black), siRNA G6PD (red) and siRNA H6PD (blue). In all cancer cell lines, silencing of either G6PD or H6PD significantly increase intracellular lactate level. Data are expressed as % vs CTR ± SD (n = 3). *p < 0.05 and ***p < 0.001 vs corresponding scramble. Panels (**c**–**e**) display the relative metabolite abundance of 6-phosphogluconic acid (6-PGluc), d-ribulose-5-phosphate (Ru5P), d-ribose-5-phosphate (R5P), xylulose 5-phosphate (X5P), sedoheptulose-7-phosphate (S7P) and d-glyceraldehyde 3-phosphate (GAD3P) in MCF-7 (**d**), MDA-MB-231 (**e**) and A549 (**f**) under control condition. Data are expressed as mean ± SD (n = 3). *p < 0.05 and ***p < 0.001 vs corresponding scramble.

In all cell lines, lactate concentration was not modified by scramble siRNA (Fig. 3a–c). By contrast, it was significantly increased by either G6PD and H6PD siRNA, suggesting that inhibition of cytosolic or reticular PPP similarly increased glycolytic rate while reducing the flux of glucose-derived carbons into mitochondria (Fig. 3a–c).

An opposite response was observed when PPP was considered. Under control conditions, MCF-7, MDA-MB-231 and A549 cells showed relatively low levels of all PPP intermediates and mostly of R5P that was virtually undetectable (Fig. 3d–f). Inhibiting either G6PD or H6PD expression decreased all levels of PPP intermediates to values close or under the threshold of our method (data not shown).

This finding seemingly indicated that H6PD silencing might impair PPP activity to a similar degree of G6PD siRNA. However, the low signal related to these metabolites prevented an accurate evaluation of this effect and the relative response to either gene silencing procedure. On the other hand, all studied cell types showed relatively high levels of d-ribose under control conditions. The only acknowledged source of this pentose is represented by R5P de-phosphorylation that might have occurred either spontaneously during sample preparation^17^ or as the result of a reaction catalyzed by the mammalian enzyme low-molecular-weight protein tyrosine phosphatase-A^18^. Regardless of the mechanism underlying R5P hydrolysis, a large literature previously documented that cell cultured under standard condition synthetize this pentose only through the oxidative and non-oxidative PPP arms^13,19^. Accordingly, we focused our attention on d-ribose to verify the effect of G6PD or H6PD silencing on PPP activity.

**Figure 4:**
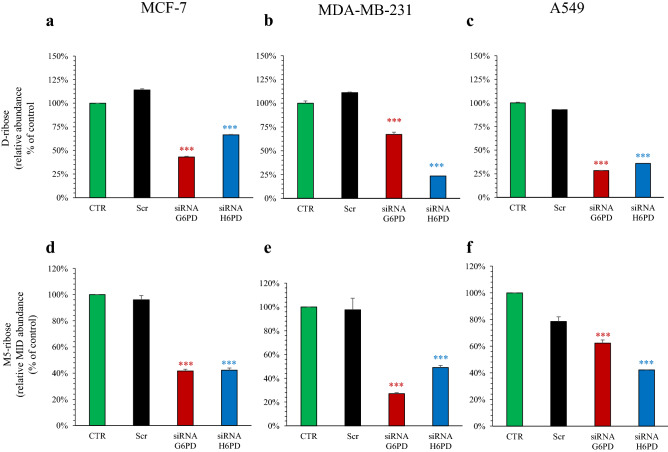
Effect of G6PD and H6PD silencing on cell content of d-ribose and M5-ribose. Relative metabolite abundance of d-ribose in MCF-7 (**a**), MDA-MB-231 (**b**) and A549 (**c**) under control condition (CTR, green), scramble siRNA (Scr, black), siRNA G6PD (red) and siRNA H6PD (blue). Scramble siRNA-transfected cells did not significantly affect d-ribose level in all cell lines. By contrast, silencing G6PD expression significantly decreased d-ribose level in all cell lines. The same response was obtained after down-regulation of H6PD. Mass isotopomer distribution (MID) of M5-ribose in MCF-7 (**d**), MDA-MB-231 (**e**) and A549 (**f**) under control condition (CTR, green), scramble siRNA (Scr, black), siRNA G6PD (red) and siRNA H6PD (blue). G6PD down-regulation significantly decreased M5-ribose content in all cell lines compared to the respective CTR. Similarly, H6PD silencing significantly reduced M5-ribose in breast cancer lines and lung adenocarcinoma cells. Data are expressed as % of CTR ± SD. ***p < 0.001 vs corresponding scramble.

In MCF-7, MDA-MB-231 and A549 cells, scramble siRNA administration did not significantly affect d-ribose level (Fig. 4a–c). By contrast, silencing G6PD expression eventually resulted in a significant reduction of the pentose amount. Intriguingly, H6PD silencing impaired to a similar extent (in MCF-7 and A549) or even more (in MDA-MB-231) pentose levels when compared to G6PD siRNA (Fig. 3a–c).

Altogether, the responses of intracellular PPP intermediates and d-ribose to gene manipulation thus suggested that inhibiting G6PD or H6PD causes a remarkably similar deceleration in overall cell PPP rate. Nevertheless, this finding seems somewhat paradoxical since it conflicts with the commonly accepted notion about the low rate of the reticular pathway^20^.

To verify this hypothesis, and thus to clarify the role of H6PD in overall hexose dehydrogenation, we cultured MCF-7, MDA-MB-231 and A549 cells, selectively silenced for either G6PD or H6PD, in the presence of glucose labeled with ^13^C in all carbon positions. We thus estimated the mass isotopomer distribution (MID) of d-ribose to depict the relative contribution of exogeneous glucose to cellular content of labeled d-ribose (M5-ribose).

Scramble siRNA administration did not affect M5-ribose MID in MCF-7 and MDA-MB-231 cells while a slight, and non-significant, decrease was observed in A549 ones (Fig. 4d–f). G6PD-siRNA significantly decreased M5-ribose content in all cell lines compared to the respective scramble siRNAs. Similarly, H6PD silencing significantly decreased M5-ribose amount even more than G6PD-siRNA in A549 cells (Fig. 4d–f).

**Figure 5:**
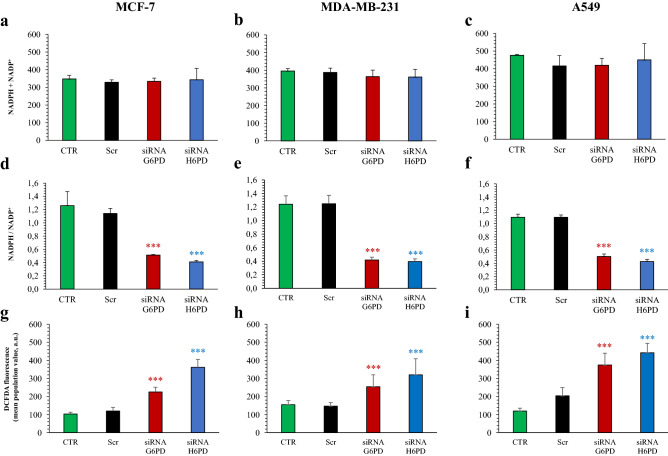
Effect of G6PD and H6PD silencing on cell redox status. Total NADPH + NADP content was similar in MCF-7 (**a**), MDA-MB-231 (**b**) and A549 (**c**) under control condition (CTR, green), scramble siRNA (Scr, black), siRNA G6PD (red) and siRNA H6PD (blue). By contrast, NAPDH/NADP^+^ ratio was significantly decreased by either G6PD or H6PD silencing in all three cell lines (**d**–**f**). This response was paralleled by an opposite behavior of ROS cell content that was instead significantly increased by both gene silencing (**g**–**i**). Data are expressed as mean ± SD. ***p < 0.001 vs corresponding scramble.

The response of overall PPP activity to gene silencing was further confirmed by the analysis of NADPH/NADP^+^ ratio. In all three cell lines, the total amount of this phosphorylated cofactor was left unchanged (Fig. 5a–c). By contrast, both G6PD and H6PD siRNA significantly decreased the NADPH/NADP^+^ ratio in all studied cell lines (Fig. 5d–f). In mammalian cells, NADPH is a crucial co-factor for both bio-reductive syntheses and for glutathione-dependent antioxidant responses. Thus, the decreased NADPH availability seems coherent with the observed reduction in proliferating activity. To verify whether decreased NADPH/NADP^+^ ratio affected the overall cell reductive power, we evaluated the presence of reactive oxygen species (ROS) by flow cytometric analysis after cellular staining with 2′,7′ dichlorodihydrofluorescein-diacetate (H_2_DCFDA). The results indicate that silencing of either glucose dehydrogenase significantly increases oxidative stress (Fig. 5g–i).

## Discussion

Present data document that inhibiting the expression of either G6PD or H6PD causes a similar decrease in cell content of PPP intermediates and d-ribose. This response is followed by a similar decrease in NADPH content, that is paralleled by increased ROS levels and followed by a deceleration in culture growth of cancer cell lines representative of breast and lung carcinoma. Differently from current notions considering the reticular pathway as characterized by a very low activity^20^, these observations indicate that H6PD channels a comparable glucose flux with respect to its cytosolic counterpart G6PD.

The PPP deceleration induced by G6PD-siRNA apparently disagrees with the acknowledged abundance of this enzyme. Indeed, previous literature reported a G6PD catalytic function in intact cells as low as 2% of its maximum rate^2,21^ measured in cell lysates at saturating substrate and NADP^+^ concentrations. However, while this notion has been described for normal cells, the high need for NADPH reducing equivalents and R5P, associated with the high proliferating activity, renders cancer cells strictly dependent upon this pathway^22^. Accordingly, the present data confirm the relevance of PPP-related glucose metabolism in preserving cancer cell proliferation, although the mechanisms underlying this link were not investigated.

On the other hand, the similar metabolic response to G6PD or H6PD gene silencing might suggest that cytosolic and reticular PPP could be independent and active on different glucose or glucose-6P pools. However, the high contribution of reticular pathway to overall rate of the PPP oxidative arm intrinsically implies an exchange mechanism able to transfer Ru5P, X5P, R5P, GAD3P and, possibly, fructose-6P from the ER lumen to the cytosol. To the best of our knowledge, this exchange has not been conclusively ascertained, although the pivotal role of glucose metabolism in ER unfolded protein response already suggested its presence^23–25^. Accordingly, the inevitable interference between reticular and cytosolic PPP most likely implies the presence of reciprocal control mechanisms that cannot be defined, based on the present data.

This concept is partially confirmed by the comparable increase in cell lactate content induced by the inhibition of either enzyme. This finding agrees with previous experiments in cancer cells: Marini et al. already reported an increased lactate release despite a normal NADH/NAD ratio and reduced glucose consumption in H6PD-siRNA cells^12^. Similarly, Benito and coworkers observed an increased lactate production, despite invariant glucose disposal, after G6PD gene silencing^14^. In the same study, this response was not reproduced by transketolase siRNA^14^. Accordingly, the interference on glycolytic flux seems a selective feature of the oxidative PPP arm. In agreement with this hypothesis, decreasing NADPH levels might enhance oxidative stress, stabilizing the Hypoxia Inducible Factor with the final consequence of a hampered pyruvate access to mitochondrial Krebs cycle^26,27^. On the other hand, the increased lactate production in H6PD-siRNA cells extends this concept, documenting that the reticular PPP contributes to the regulation of glycolytic flux even in the presence of normal G6PD levels in the cytosol.

The decrease in PPP intermediates was evident at our metabolomic approach. However, the close-to-threshold nature of this signal prevented any accurate comparison between the response to G6PD or H6PD silencing. This evaluation was instead possible when d-ribose levels were analyzed. Although the mechanisms explaining the high pentose levels cannot be defined in the present study, its decrease was nicely confirmed by the experiments with ^13^C-glucose that documented a marked decrease in ribose synthesis after gene silencing of either enzyme. In our experimental setting, d-ribose can only derive from the de-phosphorylation of R5P that represents the building block for nucleoside and nucleotide synthesis. Accordingly, the decreased abundance of this free pentose might indicate a deceleration in the synthesis rate of its phosphorylated form. In agreement with this hypothesis, G6PD- or H6PD-siRNA simultaneously decreased both d-ribose concentration and culture growth in all studied cancer cell lines. This observation confirms previous studies reporting the strict dependence of cancer cells on the high levels of NADPH and ribose equivalents for the synthesis of fatty acids and nucleic acids asked by proliferating activity^2^. However, these same data extend this acknowledged notion, since the comparable response to either gene silencing documents that G6PD alone cannot account for all activities attributed to PPP. Rather, it needs to be complemented by a correct function of its reticular counterpart H6PD.

In conclusion, the present data extend current models considering PPP as a pathway selectively located within the cytosol. Disregarding the reticular part of PPP and focusing only on G6PD function might hamper our comprehension of PPP role in cancer cell biology.

## Methods

### Cell culture

MCF-7, MDA-MB 231 and A549 cell lines was routinely grown in high glucose (25 mM) Dulbecco’s modified Eagle’s medium (DMEM) containing 4 mM l-glutamine, supplemented with 10% fetal bovine serum and with 100 U/ml penicillin and 100 µg/ml streptomycin. Cells were incubated at 37 °C in a 5% CO_2_ incubator. All reagents for media were purchased from Life Technologies (Carlsbad, CA, USA).

### Transfection assay and cell growth assay

Cells were plated in 6-well plates in standard. After 24 h, silencing of G6PD and H6PD expression was achieved by transfecting cells with either G6PD-siRNA (Silencer Select ID s5447, Thermo Fisher Scientific) or H6PD-siRNA (Silencer ID s97551, Thermo Fisher Scientific). Silencer Select Negative Control #2-siRNA (Thermo Fisher Scientific), was used as control. Transfection was performed with 40 pmol/ml siRNAs and 2.5 μL/mL Lipofectamine 2000 (Thermo Fisher Scientific). After 48–72 h of incubation cells were collected for the experiments and counted with an automated cell counter.

### Real-time PCR

Total RNA was purified by Quick RNA Miniprep Plus kit (Zymo Reseaerch) according to the manufacturer’s instructions and reverse transcribed by the SensiFAST cDNA Synthesis KIT (Bioline, Aurogene). Real-Time PCR (RT-PCR) was performed by SensiFAST SYBR (Bioline). Primers were designed by PRIMER 3 software and purchased from TIB MOLBIOL (Genoa, Italy). Primers sequences and RT-PCR conditions are available upon request.

G6PD and H6PD gene levels were normalized on the mean of three housekeeping genes: β2 microglobulin (β2), TATA-box binding protein (TBP) and glucoronidase-β (GUSB) for A549 or β2, TBP and hypoxanthine phosphoribosyltransferase 1 (HPRT) for MCF-7 and MDA-MB-231, respectively. Relative expression of target genes was calculated by Q-Gene program^28^.

### Western blot analysis

Protein extraction was performed in RIPA buffer supplemented with protease inhibitor cocktail. Western blot experiments were performed according to the standard procedure with the following antibodies: anti-G6PD and anti-β-actin (Cell Signalling, Danvers, MA, USA), anti-H6PD (Novus Biologicals).

### Cell culture growth, cell death and cell cycle analysis

Cell growth of the tumor cell line cultures was assessed by counting trypan-blue-negative cells for four days after siRNA administration. Assessment of the percentage of dead cells in the cultures was performed by propidium iodide (PI) exclusion assays. Briefly, cell cultures were stained with 3 µM Propidium Iodide (PI) and the percentage of PI-positive cells, which incorporated PI because of damaged plasma membrane, was assessed by flow cytometry (FacsCalibur, BD Biosciences, San Diego, CA). Ten thousand cells per sample were analyzed. Estimation of cell proliferation was obtained by analysis of the % of cells in the cell cycle S phase. To this end, flow-cytometric measurements of DNA content was performed on cells permeabilized with 0.05% Triton X-100, stained with 30 µg/ml PI plus 0.5 mg/ml RNase for 20 min, and measured on a FacsCalibur flow cytometer (BD Biosciences, San Diego, CA). Cell cycle phase distributions were achieved from DNA content histograms of at least 10 thousand cells.

### NADPH + NADP assay and oxidative stress evaluation

NADPH + NADP content and NADPH/NADP in cell lysates were evaluated spectrophotometrically, at 450 nm, using the NADP/NADPH Assay Kit (Abcam: ab65349), following the manufacturer’s instructions. To evaluate ROS levels, cells were stained for 10 min at 37 °C with 2′,7′-dichlorodihydrofluorescein diacetate (H_2_DCFDA) at a concentration of 1 µM (Thermo Fisher Scientific, Waltham, MA, USA). H_2_DCFDA is a non-fluorescent dye which is cleaved inside cells to 2′,7′-dichlorofluorescein (H_2_DCF). In the presence of oxidants, H_2_DFC is converted in turn to the fluorescent compound DCF. Samples were measured on a FacsCalibur flow cytometer (BD Biosciences, San Diego, CA) and the mean fluorescence value for each cell population was estimated. The analysis was confined to viable cells only, after gating based on forward- and side-scatter characteristics. Ten thousand cells per sample were analyzed.

### Metabolites extraction from cell culture samples

For untargeted experiments, cells in 6-well plates were incubated for 48 h in medium containing 25 mM, in the presence or the absence of specific siRNA.

For labelling experiments, cells were incubated for 48 h in fresh media supplemented with 25 mM [U-^13^C_6_]-Glc (Cambridge Isotope Laboratories, Inc.) in the presence or the absence of specific siRNA.

For metabolites extraction, cells were quickly rinsed with NaCl 0.9% and quenched with 500 µl ice-cold 70:30 acetonitrile–water. Plates were placed at − 80 °C for 10 min, collected by scraping, sonicated and then centrifuged at 12,000×*g* for 10 min at 4 °C. The supernatant was collected in a glass insert and dried in a centrifugal vacuum concentrator (Concentrator plus/Vacufuge plus, Eppendorf) at 30 °C for about 2.5 h. Samples were then resuspended in 150 μl of H_2_O prior to analyses.

### LC–MS metabolic profiling

The analysis was performed using an Agilent 1290 Infinity UHPLC system and an InfintyLab Poroshell 120 PFP column (2.1 × 100 mm, 2.7 μm; Agilent Technologies), coupled with a quadrupole-time of flight hybrid mass spectrometer (Agilent 6550 iFunnel Q-TOF) and equipped with an electrospray Dual JetStream source operated in negative mode.

The injection volume was 15 μL, the flow rate was 0.2 mL/min with column temperature set at 35 °C. Both mobile phases A (100% water) and B (100% acetonitrile) contained 0.1% formic acid, the injection volume was 15 μL and LC gradient conditions were: 0 min: 100% A; 2 min: 100% A; 4 min: 99% A; 10 min: 98% A;11 min: 70% A; 15 min: 70% A; 16 min: 100% A with 5 min of post-run. Flow rate was 0.2 mL/min and column temperature was 35 °C.

Mass spectra were recorded in centroid mode in a mass range from m/z 60 to 1050 m/z.

The mass spectrometer operated using a capillary voltage of 3.7 kV. Source temperature was set to 285 °C, with 14 L/min drying gas and a nebulizer pressure of 45 psig. Fragmentor, skimmer, and octopole voltages were set to 175, 65, and 750 V, respectively.

Active reference mass correction was performed through a second nebulizer using the reference solution (m/z 112.9855 and 1033.9881) dissolved in the mobile phase 2-propanol–acetonitrile–water (70:20:10 v/v). Data were acquired from m/z 60–1050. Data analysis and isotopic natural abundance correction was performed with MassHunter ProFinder and MassHunter VistaFlux software (Agilent).

### Chemicals

All chemicals and solvents used for extraction buffer and for liquid chromatography were LCMS Chromasolv purity grade. Acetonitrile, methanol, 2-Propanol and water was purchased from Honeywell, chloroform and formic acid were purchased from Sigma-Aldrich.

### Statistical analysis

All experimental group was studied in triplicate. Data are presented as mean ± standard deviation (SD). Differences among the experimental conditions were tested using analysis of variance (ANOVA), as appropriate. Statistical significance was considered for p values < 0.05. Statistical analyses were performed using SPSS software Advanced Models 15.0 (Chicago, Illinois).

## Supplementary Information

Supplementary Figure 1.

## Data Availability

Correspondence and requests for materials should be addressed to the Corresponding author, Dr. Vanessa Cossu (e-mail: vane.6291@gmail.com).
