# Supplementary material for: Two high-rate pentose-phosphate pathways in cancer cells
Source: Sci Rep. 2020 Dec 17;10:22111. doi: 10.1038/s41598-020-79185-2 (PMC7746718; doi:10.1038/s41598-020-79185-2)
Supplement: Supplementary file 1 — Supplementary Figure 1. [file 41598_2020_79185_MOESM1_ESM.pdf]

TWO HIGH-RATE PENTOSE-PHOSPHATE PATHWAYS IN CANCER CELLS

Vanessa Cossu, Marcella Bonanomi, Matteo Bauckneht, Silvia Ravera, Nicole Righi, Alberto Miceli, Silvia Morbelli, Anna Maria Orengo, Patrizia Piccioli, Silvia Bruno, Daniela Gaglio, Gianmario Sambuceti, Cecilia Marini.

Supplementary Figure Legend

Supplementary Figure 1. The full-length gels.

Panels a, b and c show the original gels reporting H6PD, G6PD and Actin expression in MCF-7 (a), MDA-MB-231 (b) and A549 (c) cells under control condition (CTR), scramble siRNA (Scr), siRNA G6PD and siRNA H6PD.

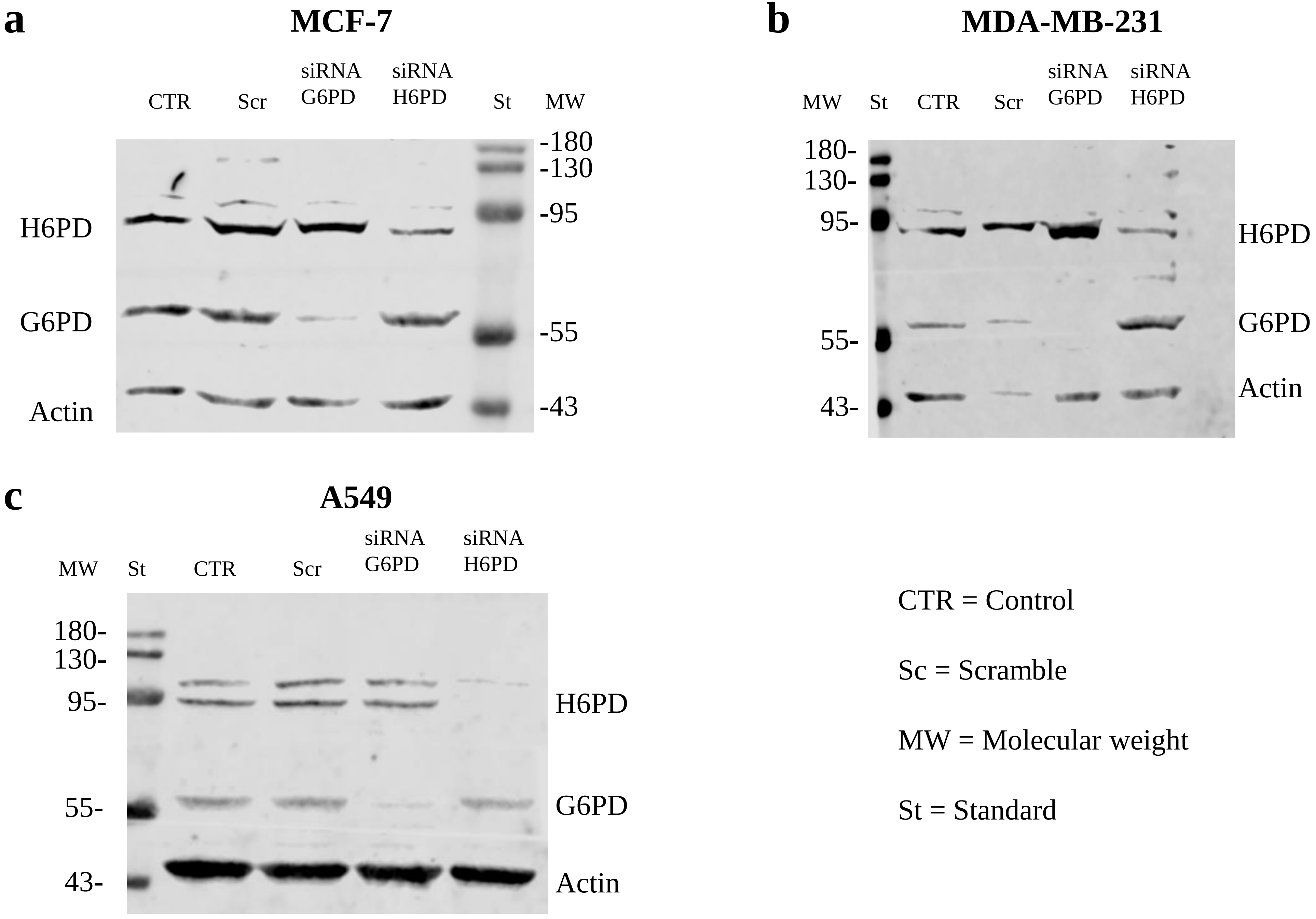
a
MCF-7
CTR
Scr
siRNA G6PD
siRNA H6PD
St
MW
-180
-130
-95
-55
-43
H6PD
G6PD
Actin
b
MDA-MB-231
MW
St
CTR
Scr
siRNA G6PD
siRNA H6PD
180-
130-
95-
55-
43-
H6PD
G6PD
Actin
c
A549
MW
St
CTR
Scr
siRNA G6PD
siRNA H6PD
180-
130-
95-
55-
43-
H6PD
G6PD
Actin
CTR = Control
Sc = Scramble
MW = Molecular weight
St = Standard
